# Traditional bullying and cyberbullying at schools in Germany: Results of the HBSC study 2022 and trends from 2009/10 to 2022

**DOI:** 10.25646/11872

**Published:** 2024-03-04

**Authors:** Saskia M. Fischer, Ludwig Bilz, for the HBSC Study Group Germany

**Affiliations:** Brandenburg University of Technology, Cottbus-Senftenberg, Department of Health

**Keywords:** BULLYING, CYBERBULLYING, PREVALENCE, DISTRIBUTION, TRENDS, SCHOOL, CHILDREN, ADOLESCENTS, VIOLENCE, HBSC, SURVEY, GERMANY

## Abstract

**Background:**

Bullying is a form of violence that is carried out repeatedly, with the intention of causing harm and with an imbalance of power between those involved. Bullying has serious negative effects on the mental health of adolescents and thus represents a significant health risk in childhood and adolescence.

**Methods:**

Based on data from the Health Behaviour in School-aged Children (HBSC) study from the survey year 2022 in Germany (N = 6,475), the prevalence of school bullying and cyberbullying among 11-, 13- and 15-year-olds in Germany was analysed. In addition, the prevalence of school bullying and cyberbullying was analysed as a trend from 2009/10 to 2022 (bullying) and from 2017/18 to 2022 (cyberbullying).

**Results:**

Around 14 % of the learners surveyed reported direct experience of bullying at school, and around 7 % reported cyberbullying experiences as bullied and/or bullying victims. Adolescents who identified as gender diverse were particularly likely to report bullying experiences. School bullying decreased over time, but remained stable between 2017/18 and 2022. Cyberbullying, on the other hand, increased in 2022 compared to 2017/18.

**Conclusions:**

Experiencing bullying at school and online is an everyday experience for many children and young people, so there is still a need for the broad implementation of effective anti-bullying measures in schools.

## 1. Introduction

Bullying is a specific form of violence that is characterised by the fact that it is carried out repeatedly and with the intention of causing harm. There is an imbalance of power between the students involved, which makes it difficult for the bullied to defend themselves against the bullying alone and without the help of others [1]. The power imbalance between students can be caused by differences in physical size and strength, for example, but also by aspects such as social integration. Acts of bullying can include insults, punches, kicks, spreading rumours or social exclusion. If the bullying is mediated by the media (e.g. via social networks or chat groups), it is called cyberbullying.

Cyberbullying is often defined analogue to school bullying as bullying in the digital space. However, the definitional aspect of repetition in particular is also repeatedly discussed (e.g. [2]). Schultze-Krumbholz and colleagues [3] also considering findings on students’ understanding of cyberbullying, suggest that cyberbullying should be understood as ‘aggressive behaviour by a person with an intention to harm or cause harm’ ([3, P. 375] translation by the author). The key characteristics of cyberbullying compared to school bullying outside the digital space are that the bullied students often do not know who is doing the bullying. This anonymity can further increase the power imbalance that exists between the bully and the bullied [4]. In addition, cyberbullying has a larger audience than school bullying and it is almost impossible for those affected to escape the bullying [4].

Despite greater social awareness of bullying and the implementation of various anti-bullying measures in many schools, bullying remains an everyday experience for many students in all types of schools [5]. This is particularly problematic because bullying can have serious negative consequences. These include, for example, academic underachievement and a higher level of school avoidance, but also higher risks of depression, anxiety, psychosomatic complaints, self-harming behaviour and suicidal tendencies [6, 7, 8]. Some of these risks are more than threefold higher as a result of bullying experiences [7, 8]. Learners who are bullied are particularly at risk. However, bullying can also have negative consequences for those who practise bullying or those who observe bullying [7, 9]. Bullying therefore represents a potential health risk for all learners.

Various studies show that cyberbullying is reported less frequently overall than school bullying outside the digital space [5, 10]. However, there is a high degree of overlap between school bullying and cyberbullying in terms of the learners involved [10, 11, 12, 13]. Just like bullying at school, cyberbullying can have serious negative consequences for those affected [10]. Some findings suggest that the risk of externalising and internalising problems after cyberbullying experiences is even higher than in the case of school bullying outside the digital space [13]. This means that both school bullying and cyberbullying are associated with high social costs, such as school avoidance and academic performance losses, as well as health restrictions and necessary therapeutic measures [6, 14].

Analyses based on data from the HBSC cycles between 2001/02 and 2017/18 suggest that bullying in schools tends to decrease over time [5]. However, the COVID-19 pandemic and the associated measures to contain the pandemic may have changed this trend, although the corresponding empirical findings are contradictory. For example, school lockdowns have prevented bullying that does not take place online [15, 16]. Some empirical findings indicate that the incidence of bullying (both school bullying and cyberbullying) initially decreased with the school lockdowns [15, 16, 17], but increased again with the resumption of face-to-face teaching in schools [15]. However, the incidence of bullying remained below the pre-pandemic level [15]. It is possible that organisational changes, such as learning in smaller classes and increased individual support for learners by teachers, but also social effects (e.g. more cohesion due to the pandemic-related crisis experience), contributed to a reduction in the incidence of bullying during the pandemic [18]. At the same time, the finding that bullying decreased after the school lockdowns could also be a short-term effect. Changes in organisational measures were not permanent and the feeling of social connectedness may have decreased rather than increased as the pandemic progressed.

Studies from regions where there were only brief or partial school lockdowns suggest that bullying increased after the acute phase of the pandemic [19].

In contrast to school bullying, there are no findings on the development of cyberbullying in Germany compared to previous survey cycles from the HBSC study, as cyberbullying experiences were surveyed for the first time in 2017/18 [5]. Possible developments between the 2017/18 and 2022 survey cycles could also be related to the pandemic-related experiences of learners. With regard to the influence of the COVID-19 pandemic on cyberbullying experiences, international research shows very heterogeneous results. Some studies show a significant decline in cyberbullying in connection with school lockdowns [15], while others show a decline that is significantly weaker than the decline in traditional bullying [18]. Still others report an increase in cyberbullying during the pandemic [20]. Online teaching during the COVID-19 pandemic may have led to school bullying occurring exclusively in the form of cyberbullying. During the COVID-19 pandemic, the daily use of online media among adolescents has increased further [21, 22]. Daily use of digital media, and in particular frequent social interaction in the digital space, can increase the risk of bullying (both traditional and online) [23]. As social contact with peers was almost exclusively possible digitally for adolescents due to pandemic-related contact restrictions, there may therefore have been an increase in cyberbullying in the 2022 survey cycle.

This article will first analyse the prevalence of bullying and cyberbullying in Germany in 2022. For this purpose, both the overall group of learners surveyed and various subgroups differentiated by gender, age and type of school will be analysed. In addition, the question of how the prevalence of bullying and cyberbullying has changed from 2009/10 and 2017/18 to 2022 will be analysed.

**HBSC 2022**

**Data holder:** HBSC Study Group Germany

**Objective:** The aim of the study is to analyse the health and health behaviour of students. Continuous health monitoring through the HBSC study contributes to informing decision-makers in policy and practice about the current fields in prevention and health promotion in childhood and adolescence. A particular focus is on the influencing factors and the social contexts of health in the young generation.

**Study design:** Cross-sectional survey by written questionnaire every four years

**Population:** Students with average ages 11, 13, and 15

**Sampling:** Observation units are schools and the class groups clustered within them. From the population of all state general education schools in Germany, a cluster sample was drawn. In order to obtain a representative estimate (close to the distribution of the population), school size and the percentage distribution of students were included in the sampling, stratified by school type and federal state (Probability Proportional to Size (PPS) design).

**Data collection period:** March – November 2022

**Sample size:**

**2022:** 6,475 students

**All four survey cycles (2009/10 – 2022):** 21,788 students

**HBSC survey cycles:**

**Included in the articles in this issue of the Journal of Health Monitoring:**

▶ 2009/10
▶ 2013/14
▶ 2017/18
▶ 2022

More information can be found at https://hbsc-germany.de/ (German)

## 2. Methods

### 2.1 Sample design and study implementation

The Health Behaviour in School-aged Children (HBSC) study is designed as a cross-sectional study that takes place every four years in a school setting and surveys students aged around 11, 13 and 15 (mean deviation of 0.5 years). In Germany, these age groups are mainly represented in grades 5, 7 and 9. Students at general education schools in all 16 federal states in Germany have been surveyed in the school years 2009/10, 2013/14, 2017/18 and in the calendar year 2022 as part of the HBSC study. The schools approached for participation were drawn as a cluster sample from the population of all state general education schools in Germany. In order to obtain a representative estimate (close to the distribution of the population), school size and the percentage distribution of students were included in the sampling, stratified by school type (Probability Proportional to Size (PPS) design).

The HBSC study is conducted by means of a questionnaire which students complete themselves. The study has been approved by the responsible ministries or state education authorities in all federal states (except North Rhine-Westphalia, as the decision of participation lies within the schools in this federal state).

Four survey cycles of the HBSC study Germany were analysed for the present study. In addition to the current survey in 2022 (n = 6,475), three further surveys were included in the following school years: 2009/10 (n = 5,005), 2013/14 (n = 5,961) and 2017/18 (n = 4,347). All data sets were standardised and adjusted by the international HBSC consortium so that the age groups are comparable. The data collection in 2022 took place after the pandemic-related school lockdowns, when teaching in schools largely took place without pandemic-related protective measures. Further information on the HBSC study and the methodology can be found in the article by Winter & Moor [24] in this issue of the Journal of Health Monitoring.

### 2.2 Sample

**Sample 2022:** Data from N = 6,475 students at 174 schools are available from the 2022 survey cycle (50.3 % girls, 47.5 % boys, 1.7 % adolescents who identify as gender diverse; see Winter & Moor [24] for further information on the sample).

**Sample 2017/18:** N = 4,347 adolescents participated in the HBSC study 2017/18 (see Winter & Moor [24]). Further information on the methodology of the 2017/18 HBSC survey cycle can be found in Moor et al [25]; further information on the distribution of bullying in Germany in 2017/18 can be found in Fischer et al [5].

**Sample 2013/14:** Data from N = 5,818 students from the 2013/14 survey year are used for the trend analyses. The total sample from the 2013/14 survey year is larger than the sample used for the trend analyses (total sample 2013/14: N = 5,961, cf. Winter & Moor [24]) because, in contrast to the other survey cycles, special schools were also surveyed in the 2013/14 HBSC survey cycle. Various studies suggest that the incidence of bullying is particularly high at special schools [26, 27]. In order to ensure comparability with the other survey cycles with regard to the prevalence of bullying, the data from students at special schools were removed from the trend analyses (n = 143 students at ten special schools). Further information on the 2013/14 survey cycle and the incidence of bullying in 2013/14 can be found in Bucksch et al. [28] and Oertel et al. [29].

**Sample 2009/10:** Data from N = 5,005 students are available from the 2009/10 survey year (see Winter & Moor [24]). Further information on the methodology of the 2009/10 HBSC survey cycle and the incidence of bullying in 2009/10 can be found in Kolip et al. [30] and Oertel et al. [31].

A weighting factor was created for all survey cycles to ensure nationwide sample representativeness. This equalises different participation rates in the federal states and school types so that the distribution corresponds to the population. Due to the weighting, all three age categories and the binary gender categories of girls and boys are included in the analyses in equal parts from the 2017/18 survey cycle onwards. In the 2022 HBSC survey cycle, gender was not recorded exclusively in binary form for the first time, with 1.7 % of respondents indicating the category gender diverse. This was taken into account in the weighting of the 2022 data, while girls and boys were weighted equally (49.2 % each; participants who did not specify their gender were excluded). Further details on the weighting of the data can be found in the article by Winter & Moor [24].

### 2.3 Survey instruments

#### Bullying at school and cyberbullying

**Bullying at school:** Experiences of bullying at school as the bullied and the bully were assessed using two items from the Revised Olweus Bully/Victim Questionnaire (OBVQ) [32]. Students were asked how often they had ‘participated in bullying at school in the last few months’ and how often they had been ‘bullied at school in the last few months’. The response options were (1) ‘I have not bullied anyone at school in the last few months’ or ‘I have not been bullied at school in the last few months’, (2) ‘1 or 2 times’, (3) ‘2 to 3 times a month’, (4) ‘about once a week’ and (5) ‘several times a week’. The two items were dichotomised for the analysis. In order to take the repetitive aspect of bullying into account, all answers from ‘2 to 3 times a month’ (answer options 3 to 5) were classified as experiences of being bullied or bullied. Students who reported regular bullying experiences for both items were assigned to the double role of both bullying others and being bullied. The survey and categorisation were identical in all survey cycles considered (2009/10, 2013/14, 2017/18, 2022). Complete information on their bullying experiences is available from n = 5,793 students from the survey year 2022.

**Cyberbullying:** Experiences with cyberbullying were recorded in a similar way to experiences with bullying at school. The students were asked how often they had ‘bullied someone online’ or been ‘bullied online’ in the last few months. They were given the following examples: ‘e.g. you have written mean messages, emails, text messages or noticeboard postings, created websites to make fun of someone or posted or sent unflattering photos of someone without permission’ (wording in the item on the experience of being bullied analogue). The response options were collected and the responses categorised as described for school bullying. Cyberbullying was surveyed identically in the 2017/18 and 2022 survey cycles. Cyberbullying was not surveyed in the previous survey cycles. Complete information on cyberbullying experiences is available from n = 5,706 students from the 2022 survey year.

**Typology of (cyber)bullying experiences:** In order to be able to analyse the bullying experiences, a typology of bullying experiences was formed from the dichotomised items separately for school bullying and cyberbullying. This distinguishes between four categories: Uninvolved, bullied, bullies and double role bully and bullied (i.e. students who are both bullied and bully others).

**Infobox**

**Bullying**

The students surveyed were presented with an age-appropriate definition of bullying, which included the core elements of repetition, power imbalance and intent to harm. Specifically, the bullying definition was:

*We say that a person is bullied when another person or group of people repeatedly says or does mean or unkind things to him or her. It is also bullying when a person is teased with things they don’t like or deliberately excluded. The person who bullies has more power than the person being bullied and wants to harm them. Bullying does not occur when two people of equal power argue or fight with each other.*

#### Control variables

Gender, age and type of school are considered as control variables in the analyses. Gender was recorded in the 2022 survey year using the three options ‘girl’, ‘boy’ or ‘diverse’. In the previous survey cycles, gender was recorded in binary form (girl, boy). For the trend analyses, participants who did not specify their gender or classified themselves as diverse were excluded from the gender-specific analyses. The age was determined at the time of the survey using the information provided by the students on their month and year of birth and summarised with a deviation of +/- 0.5 years into the age categories ‘11 years’, ‘13 years’ and ‘15 years’.

The school type was recorded by the survey team using the school data. The respective school types within the federal states were divided into six categories in the 2022 survey year: Primary schools, secondary general schools, intermediate schools, grammar schools as well as the groups of comprehensive schools etc. (different types of comprehensive schools in the different federal states in Germany, i.e. schools in which different graduations can be obtained) and secondary schools etc. (secondary schools/combined secondary general and intermediate schools/state general education schools/intermediate schools). Special schools were not included in the sample in the 2022 survey year.

### 2.4 Statistical methods

The prevalence of bullying and cyberbullying in the survey year 2022 was calculated using the typology described for school bullying and cyberbullying. Group differences by gender, age and type of school were determined for both typologies using chi-square tests with post-hoc analyses.

To provide an overview of the development of bullying prevalence over the four survey years between 2009/10 and 2022, the percentages of the four bullying roles (typology) considered are first reported for all survey years, with group differences between survey years examined using a chi-squared test and post-hoc analyses. The trend was then analysed using logistic regression analyses with robust standard errors (maximum likelihood estimation with robust standard errors, MLR), including the control variables of gender and age. The robust standard errors were used to consider the non-normal distribution and dependence of the data within survey periods, classes and schools. In the regression analyses, one category of the typology was compared with all other categories. Predictors in the regression analyses were year of survey (dummy coded), age (dummy coded) and gender (binary; adolescents who identified as gender diverse in 2022 were excluded from the trend analyses) as well as the interaction effects of year of survey and age or year of survey and gender. These interaction effects were used to analyse whether the trend might be different for each gender or age group.

The analyses were carried out with SPSS 29 and Mplus 8.10. The significance level in the analyses of data from the 2022 survey year is p < 0.05. An alpha error correction was applied to individual comparisons. A more conservative significance level of p < 0.001 was chosen for the logistic regression analyses looking at trends in bullying at school, in order to avoid interpreting random results in many individual comparisons. As data for cyberbullying is only available from two survey years and hence the number of individual comparisons is lower than for school bullying, the conservative significance level was only chosen for the regression analyses on school bullying. For the regression analyses on cyberbullying, the significance level was set at p < 0.05.

## 3. Results

### 3.1 Prevalence of bullying and cyberbullying at school in 2022

The prevalence of school bullying and cyberbullying in the total sample and by subgroup is shown in Table 1. In 2022, most students reported that they had not been directly involved in school bullying or cyberbullying, with cyberbullying being reported even less frequently than school bullying (uninvolved school bullying: 86.1 %; uninvolved cyberbullying: 92.9 %). Among those directly involved in school bullying, most reported being bullied by others (8.6 %). Fewer reported having bullied others at school (3.4 %). With regard to cyberbullying, however, of those directly involved in bullying, roughly the same number of students reported having been bullied online (3.0 %) and having bullied others online (2.7 %). The group of those who have both been bullied and bullied others is the least represented in both school bullying (1.9 %) and cyberbullying (1.4 %).

Involvement in bullying and cyberbullying varies according to the gender of the respondent. Young people who identify as gender diverse were significantly more likely than girls and boys to report having been bullied at school or online. Girls were less likely than boys to report having been bullied at school or online. With regard to cyberbullying, girls were also less likely than young people who identify as gender diverse to have bullied others. For both school bullying and cyberbullying, girls were the least likely and youth who identified as gender diverse were the most likely to report both being bullied and bullying others (double role bully and bullied) (Table 1).

The bullying experiences of the children and adolescents surveyed differed only slightly by age. For bullying at school, 13-year-olds were more likely than 15-year-olds to report direct experiences of bullying and more likely than 11-year-olds to report having bullied others at school. With regard to cyberbullying, there were no differences between 11-, 13- and 15-year-olds’ involvement in bullying.

In terms of school type, there are many differences between the six school types and the four bullying roles analysed. Students in grammar schools were less likely than students in most other school types to have been directly involved in bullying or cyberbullying at school. The experience of being bullied by others in a school context was about equally common among students in all types of schools, while students in secondary general schools were significantly more likely to report being bullied online. In terms of bullying, students in intermediate schools were more likely than students in grammar schools and secondary general schools to report having bullied others at school. Students in grammar schools were less likely than those in comprehensive schools and secondary schools to report having bullied others online. Students who have both been bullied and bullied others at school or online are less likely to be found in grammar schools than in other types of school, although there are no differences between all types of school. All significant group differences are shown in Table 1.

### 3.2 Trend in prevalence of bullying: general trend

Table 2 shows the prevalence of bullying in the school context in the survey years 2009/10, 2013/14, 2017/18 and 2022. The comparison without the control variables of age and gender shows that overall more direct experiences of bullying were reported in 2009/10 and 2013/14 than in 2017/18 and 2022. Regarding the experience of being bullied, the information does not differ between the four survey cycles considered. However, more students reported having bullied others in 2009/10 and 2013/14 than in 2017/18 and 2022. There are no significant differences between 2009/10 and 2013/14. The survey years 2017/18 and 2022 differ only in the proportion of students who reported both being bullied and bullying others: the proportion of the double role bully and bullied is higher in 2022 than in 2017/18. However, there are no differences for this bullying role compared to the previous survey years 2009/10 and 2013/14. Due to the different weighting of the data in 2009/10 and 2013/14 compared to 2017/18 and 2022 (see section 2.2), a direct comparison of the percentages must be made with caution. Even if there are usually only small shifts due to the weighting, it is more reliable to compare the survey periods considering age and gender (see section 3.3).

Table 2 compares experiences of cyberbullying in the two survey years 2017/18 and 2022. It can be seen that students are more often represented in all cyberbullying roles in 2022 than in 2017/18.

### 3.3 Trends in the prevalence of bullying taking into account age and gender

In addition to the univariate trends considered in section 3.2, the analysis of bullying trends from 2009/10 to 2022 also takes into account the control variables of age and gender in more complex statistical analyses. This makes it possible to determine whether there are different trends for boys and girls or for younger and older adolescents. This is done by including interaction terms between age and survey year or between gender and survey year.

The results for the prevalence of bullying at school from 2009/10 to 2022 are shown in Table 3. Similar to the results of the univariate trend analysis (see Table 2), it can be seen that there is no difference in the prevalence of bullying between 2017/18 and 2022. The difference between 2017/18 and 2022 regarding the double role of bully and bullied (Table 2) disappears when the control variables are considered. However, in the earlier survey years 2009/10 and 2013/14, significantly more students reported that they had been involved in bullying, and in particular that they had bullied others, compared to 2022.

The trend is comparable for girls and boys (not shown in the table; all confidence intervals include 1 for all bullying roles, p > 0.05). Looking at the trend as a function of age, it can be seen that especially the youngest respondents (compared to the oldest respondents) were not involved in bullying in 2009/10 (compared to 2022) (interaction term 11 years x year 2009/10 for the bullying role of those not involved, reference: 15-year-olds in the survey year 2022; OR = 1.8, p < 0.001, 95 % CI: 1.3 – 2.5). The increase in those not involved in school bullying in 2022 was greater among 15-year-olds than among 11-year-olds compared to 2009/10. There were therefore more uninvolved 15-year-olds in 2022 than in 2009/10. However, this is due to the fact that 11-year-olds already reported less active involvement in bullying at school in the 2009/10 survey year (uninvolved 11-year-olds 2009/10: 84.3 %, 2022: 86.1 %; uninvolved 15-year-olds 2009/10: 79.3 %, 2022: 87.8 %). A further decrease in bullying experiences since 2009/10 was therefore less possible for the group of 11-year-olds, as the youngest students surveyed have been involved in very little bullying at school since the beginning of the nationwide HBSC surveys. There are no further developments in the trend according to age group (other significant interaction terms between age group and survey year).

Table 4 shows the results of the logistic regression analyses for participation in cyberbullying in the survey years 2017/18 and 2022. Considering the control variables, it can be seen that the surveyed students reported more cyberbullying experiences in 2022 than in 2017/18. Looking at the three groups of those directly involved, a significant increase can only be observed in the double role of bully and bullied. Here, the inclusion of the control variables leads to different results than in the univariate analysis in Table 2.

There are no age differences in participation in cyberbullying in 2022. Changes between 2017/18 and 2022 are also largely uniform across age groups (interaction terms between age group and survey year). Only 13-year-olds report less involvement as bullies in cyberbullying in 2017/18 than in 2022 compared to 15-year-olds (interaction term 13 years x survey year 2017/18 for the role of bullies, reference: 15-year-olds in survey year 2022; OR = 0.4, p < 0.05, 95 % CI: 0.2 – 1.0). This means that 13-year-olds had a greater increase in experience of bullying others online between 2017/18 and 2022 than 15-year-olds. However, it should be noted that 15-year-olds were already more likely to report having bullied others online in 2017/18 (13-year-olds bullied online in 2017/18: 0.9 %, 2022: 2.9 %; 15-year-olds bullied online in 2017/18: 2.4 %, 2022: 3.2 %; see Table 1 and [5]).

A closer look at the differences between 2017/18 and 2022 by gender shows that in 2022, girls were less likely than boys to be involved in cyberbullying (and especially less likely to bully others online). In addition, the increase in cyberbullying experiences from 2017/18 to 2022 was also lower for girls than for boys (interaction term girls x survey year 2017/18 for the role of uninvolved, reference: boys in survey year 2022; OR = 0.6, p < 0.01, 95 % CI: 0.4 – 0.9; interaction term girls x survey year 2017/18 for the role of bullied; OR = 2.1, p < 0.01, 95 % CI: 1.0 – 4.3). The proportion of girls not involved in cyberbullying decreased only slightly (2017/18: 95.9 %, 2022: 94.9 %; see Table 1 and [5]), while the proportion of boys not involved in cyberbullying decreased significantly in 2022 compared to 2017/18 (2017/18: 96.1 %, 2022: 91.5 %; see Table 2 and [5]). In particular, the risk of boys bullying others online has increased (bullying boys 2017/18: 1.6 %, 2022: 4.1 %; bullying girls 2017/18: 1.0 %, 2022: 1.3 %; see Table 2 and [5]).

## 4. Discussion

### 4.1 Prevalence of bullying at school and cyberbullying in 2022

Just under 14 % of the young people surveyed said that they had been bullied and/or had bullied others at school in 2022. This means that around one in seven learners have experienced direct bullying in 2022. Given that bullying can have a negative impact not only on these directly affected learners, but also on all those who observe and experience bullying in their classrooms (see section 1), this finding underlines that bullying continues to be an everyday problem for many children and young people.

In line with current research [5, 10], the results of the HBSC 2022 survey show that cyberbullying is less commonly reported than bullying at school. In this study, only just over 7 % of students reported being bullied online and/or having bullied others online. This means that bullying experiences at school are twice as common as bullying experiences explicitly attributed to the digital space.

In the 2022 HBSC study in Germany, boys were more likely than girls to report having bullied others at school or online. More frequent bullying by boys than girls is often reported in studies [10], so the findings are consistent with the current state of research. In contrast, there is little evidence about young people who identify as gender diverse. It is known that sexual minority (LGBTQ) learners are at higher risk of being bullied [33]. In contrast, the group of adolescents who do not identify with a binary gender group has rarely been analysed separately. Studies from the USA and Finland suggest that non-binary and transgender youth are at higher risk of being bullied at school and are also more likely to be bullied themselves [34, 35]. A study from Finland suggests that students may pass on their own experiences of victimisation through bullying, or that bullying may result from inappropriate strategies for coping with internal stress and strain [35]. In general, however, it should be noted that there is a paucity of research on gender diverse youth in relation to bullying and, in particular, perpetration of bullying is rarely included. Findings from Germany appear to be particularly scarce: a systematic review of 2023 identified 111 empirical publications on bullying and LGBTQ+, none of which were from Germany [33]. There is therefore a need for further research in this area, as well as to review and categorise the available evidence.

There were few age differences in the reported incidence of bullying in 2022. 13-year-olds were more likely than the younger and older age groups to report directly experiencing bullying and bullying others. However, these differences are small and do not affect the experience of being bullied themselves. There were no age differences in cyberbullying. This contradicts the findings of the 2017/18 HBSC study [5], in which 15-year-olds were more likely than younger students to report bullying others in a school context and online. At that time, 13-year-olds were also more likely than 15-year-olds to report being bullied at school [5]. It is possible that younger adolescents are now becoming more involved in bullying themselves in an apparent attempt to protect themselves from being bullied. The results of the HBSC 2022 study suggest that bullying is more likely to be directed at younger students, although these findings are not significant. Age differences in the three age groups described have not been widely investigated and should be further explored in future studies.

An analysis of the prevalence of bullying by school type shows that bullying occurs in all types of school. Students in grammar schools tended to report less bullying in 2022 than students in other types of school. Students in secondary general schools were particularly likely to report being bullied online. However, the experience of being bullied themselves was reported in all school types. This is in line with previous studies [5, 29] and shows that bullying prevention and intervention policies are important in all types of schools.

### 4.2 Trends in the prevalence of bullying at school and cyberbullying

The analysis of the prevalence of bullying in the school context from 2009/10 to 2022 shows that less bullying was reported in 2022 than in 2009/10 and 2013/14. However, there are no differences compared to the survey year 2017/18. An increase in the double role of bullies and bullied, which seemed relevant when looking at the percentages in the survey years, is no longer evident when the control variables of age and gender are taken into account. This means that overall it can be assumed that the prevalence of bullying has remained stable since 2017/18. Compared to previous years (up to 2013/14), bullying at school has decreased, but there does not seem to have been a further decrease. This has already been mentioned in the analyses of the 2017/18 HBSC study in Germany [5]. Overall, the trend is comparable for the age groups analysed and for boys and girls. Only 11-year-olds show a smaller decrease in bullying at school between 2009/10 and 2022, but this is mainly due to the fact that the 11-year-olds surveyed in 2009/10 already reported fewer active bullying experiences than other students. A decrease in bullying experiences is only evident where there was already more bullying, so overall it could be a floor effect. This means that there is a low level of bullying at which further reductions in bullying are statistically difficult to prove. However, it should also be taken into account that the lower bullying figures in 2009/10 and 2013/14 are mainly due to the role of the bullies. This could also be a reporting effect: Learners in 2017/18 and 2022 may be more likely to refrain from reporting their own bullying behaviour as a result of increased awareness-raising activities. The reduction in bullying behaviour would then be reflected in studies, but not in everyday school life. It is also possible that students who bully others despite increased anti-bullying measures will bully different other students. In this case, the number of bullies would decrease compared to previous survey years, but not the number of bullying incidents.

Due to the COVID-19 pandemic, the question of the development of the prevalence of bullying from 2017/18 to 2022 is particularly relevant. However, the available evidence suggests that the pandemic has not led to significant changes in the prevalence of bullying in schools. It should be noted, however, that most of the pandemic-related safeguards had expired by the time of the 2022 survey. Extensive school closures due to the pandemic occurred mainly in 2020. Possible shifts in the incidence of bullying from the school context to the cyber context [15, 16], contextual amplification of bullying processes due to a general sense of insecurity [36], or contextual protective factors such as an increased sense of community or learning in smaller classes [18], as suggested by some researchers, may have taken place but would no longer be measurable in 2022. A study conducted in the US suggests that there may have been a comparatively rapid increase in school bullying after schools reopened, although this initially remained below pre-pandemic levels [15]. However, as time elapsed between the interventions and the reopening of schools, the dynamics of bullying may have adjusted so that there are no differences in the incidence of bullying compared to pre-pandemic levels. This means that the lack of change in the prevalence of bullying in schools between 2017/18 and 2022 does not rule out an influence of the pandemic on the incidence of bullying.

However, the prevalence of cyberbullying has increased. The increase mainly affects 13-year-old students and boys. The time that children and young people spend with online media continued to increase in 2022 [21, 22], which may also have been facilitated by the pandemic-related experiences in the online space (online lessons, online social contacts). Cyberbullying experiences may have increased as a result, independent of school bullying.

As bullying at school has not decreased, but cyberbullying has increased, the overall problem of bullying is greater in 2022 than in 2017/18. However, it is unclear whether more students are affected by bullying overall, or whether more students who have experienced bullying at school are now directly experiencing cyberbullying [11]. Whether there is a further increase in cyberbullying will have to be analysed in subsequent cycles of the HBSC study, as the comparison between the two survey years 2017/18 and 2022 cannot yet describe a trend.

### 4.3 Limitations

When interpreting the data on the prevalence of bullying at school and cyberbullying, it is important to bear in mind the way in which bullying experiences were collected. For example, for the school bullying survey, children and young people were asked about their experiences of bullying ‘at school’. For the cyberbullying survey, they were explicitly asked about their experiences online. It is possible that students thought of bullying experiences that included both contexts (e.g. cyberbullying in a class chat during the school day), which could have led to double counting. In addition, children and adolescents may make less of a distinction between offline and online spaces, meaning that bullying that was not explicitly categorised as cyberbullying by the adolescents did not necessarily take place outside of the online space. This means that cyberbullying may have been underreported.

The survey on bullying and cyberbullying at school was self-reported by the adolescents, so social desirability may have led to underreporting of bullying (and especially bullying practice, see section 4.2). Completing the questionnaire in class may also have influenced students’ reporting behaviour (e.g. reinforcement of social desirability; fear that classmates will see their own statements). In addition, experiences of bullying (school bullying and cyberbullying) were measured with only a few items (one item each for experiences of being bullied and bullying in school and cyberbullying). This could also lead to an underestimation of bullying, as learners may not directly recall their own experiences of bullying as such in a global item. Including specific types of bullying with examples could lead to more accurate information.

Overlaps between experiences of school bullying and cyberbullying were not considered in the present analyses. In addition, the chosen typology may be an over-simplified representation of reality, as studies indicate that experiences of cyberbullying in particular are rarely made in one of the differentiated roles [37, 38].

The trend was analysed considering the control variables of age and gender. The trend might be different for other aspects such as type of school or migration background. In addition, future trend analyses should also include adolescents who identify as gender diverse, as the findings for 2022 show that the bullying experiences of these students differ from those of adolescents with binary gender identification (as girls and boys).

Possible effects of the COVID-19 pandemic on the incidence of bullying among students can only be analysed indirectly through comparisons over time. These time comparisons, as here between the 2017/18 and 2022 survey cycles, are distorted by other influences. Changes cannot be attributed solely to the pandemic, but are inextricably linked to other temporal and contextual influences.

### 4.4 Conclusions

This study shows that bullying is still an everyday experience for many children and young people. Compared to previous years, less bullying was reported at school in 2022. Compared to 2017/18, there was no increase, but also no further decrease in school bullying. However, more experiences of cyberbullying were reported in 2022 than in 2017/18, so there may have been an overall increase in bullying. However, it is unclear whether the number of students affected by school bullying and cyberbullying has increased, or whether more students affected by school bullying are also directly experiencing cyberbullying. This will require further statistical analysis. In addition, the development of the prevalence of bullying needs to be further monitored and analysed in the coming years.

Overall, the continuation and further implementation of anti-bullying policies in schools is necessary to successfully counteract bullying. In addition to the students themselves, appropriate interventions should also target teachers and the school system as a whole. Teachers should be provided with a range of successful anti-bullying strategies to choose from depending on the situation, and they should be encouraged to be confident in their own pedagogical behaviour even in the face of bullying incidents. Collaborative strategies appear to be particularly promising [39, 40], so collaborations within and outside the school should be established and utilised. This includes collaboration with local health services (e.g. counselling services, doctors, psychotherapists, clinics), as bullying poses a significant risk to young people’s physical and mental health. Students in the classroom should be encouraged to stand by the bullied students, thus depriving the bullied students of motivating positive feedback (bystander behaviour; see participant role approach) [41, 42]. Appropriately trained and available school social workers can help to implement school-wide anti-bullying policies, thereby reducing the burden on teachers and acting as a resource for students.

In particular, the prevalence of cyberbullying needs to be monitored and taken into account and analysed in later cycles of the survey in order to identify and counteract possible negative effects on students. In this context, efforts by society as a whole to help young people become competent in the use of digital media and appropriate social digital communication are also relevant. This includes both in-school and out-of-school promotion of media literacy as well as promotion of social skills and conflict resolution skills. In addition, young people need to have someone they can talk to in confidence if they have negative experiences online, are bullied at school or online, or witness bullying. Appropriate youth work services and parental counselling are just as important as legal measures to protect children and young people in the digital space.

## Key statement

- As part of the HBSC study, almost 6,500 students in grades 5, 7 and 9 were asked about their experiences with bullying and cyberbullying.
- In 2022, just under 14 % of adolescents stated that they had experienced bullying at school.
- Just under 7 % of students reported experiences with cyberbullying in 2022.
- Adolescents who identified as gender diverse were significantly more likely to report having been bullied at school or online.
- There are no significant differences between the survey years 2017/18 and 2022 in terms of the prevalence of bullying at school, but cyberbullying has increased.

## Figures and Tables

**Table 1:** Bullying experiences by gender, age category and type of school in relation to experiences with school bullying and cyberbullying in the survey year 2022 (school bullying: n = 2,942 girls, n = 2,727 boys, n = 98 gender diverse; cyberbullying: n = 2,913 girls, n = 2,669 boys, n = 98 gender diverse) Source: HBSC Germany 2022

Uninvolved (in %)		Suffered bullying (in %)	Bully (in %)	Double role bully and bullied (in %)
**School bullying**				
**Total (N = 5,793)**	**86.1**	**8.6**	**3.4**	**1.9**
**Gender** (X^2^ (6) = 97.4, p < 0.001, V = 0.09, n = 5,767)				
Girls (n = 2,942)	88.0_a_	8.9_d_	2.0_f_	1.1_h_
Boys (n = 2,727)	84.8_b_	7.8_d_	4.9_g_	2.5_i_
Gender diverse (n = 98)	65.7_c_	23.5_e_	3.9_f, g_	6.9_j_
**Age** (X^2^ (6) = 18.7, p = 0.005, V = 0.04, n = 5,736)				
11 years (n = 1,862)	86.2_k,l_	9.3_m_	2.6_n_	1.9_p_
13 years (n = 1,937)	84.2_l_	9.3_m_	4.3_o_	2.3_p_
15 years (n = 1,937)	87.8_k_	7.4_m_	3.4_n,o_	1.4_p_
**Type of school** (X^2^ (15) = 52.8, p < 0.001, V = 0.06, n = 5,680)				
Primary school (n = 249)	79.4_q_	13.2_s_	5.1_t, u_	2.2_v, w, x_
Secondary general school (n = 154)	88.4_q, r_	7.4_s_	1.3	2.8
Intermediate school (n = 544)	85.1_q_	9.4_s_	3.6_t, u_	1.8_v, w, x_
Grammar school (n = 3,075)	88.8_r_	7.4_s_	2.7_u_	1.1_w_
Comprehensive school etc. (n = 672)	84.0_q_	10.0_s_	3.5_t, u_	2.5_v, x_
Secondary school etc. (n = 1,099)	83.1_q_	9.0_s_	5.6_t_	2.3_v, w, x_
**Cyberbullying**				
**Total (N = 5,706)**	**92.9**	**3.0**	**2.7**	**1.4**
**Gender** (X^2^ (6) = 99.9, p < 0.001, V = 0.09, n = 5,679)				
Girls (n = 2,913)	94.9_a_	3.1_d_	1.3_f_	0.8_h_
Boys (n = 2,669)	91.4_b_	2.6_d_	4.1_g_	1.8_i_
Gender divers (n = 98)	77.7_c_	11.7_e_	4.9_g_	5.8_j_
**Age** (X^2^ (6) = 14.5, p = 0.025, V = 0.04, n = 5,652)				
11 years (n = 1,817)	94.0_k_	3.0_l_	2.0_m_	1.0_n_
13 years (n = 1,914)	92.6_k_	3.4_l_	2.9_m_	1.1_n_
15 years (n = 1,921)	92.3_k_	2.6_l_	3.2_m_	1.9_n_
**Type of school** (X^2^ (15) = 73.1, p < 0.001, V = 0.07, n = 5,706)				
Primary school (n = 239)	89.3_o, p, q_	4.6_r, s_	2.3_t, u_	3.8_v_
Secondary general school (n = 149)	91.5_q_	6.5_s_	1.6_t, u_	0.4_w, x_
Intermediate school (n = 536)	93.6_o, p, q_	2.5_r_	2.8_t, u_	1.1_v, w, x_
Grammar school (n = 3,054)	95.1_p_	2.5_r_	1.6_u_	0.8_x_
Comprehensive school etc. (n = 658)	91.0_o, q_	2.8_r_	4.0_t_	2.1_v, w_
Secondary school etc. (n = 1,070)	90.9_o, q_	2.9_r_	3.9_t_	2.4_v, w_

Subscripts indicate subgroups that are not significantly different in the post-hoc analyses. Subgroups that do not have the same letter within a bullying role are therefore significantly different from each other. In the post-hoc analyses, the alpha errors were adjusted according to Bonferroni (p_gender and age_ < 0.017; p_school type_ < 0.003). Values slightly above or below 100 % are due to rounding of decimals. The number of cases (n) refers to the number of cases before weighting. All percentages are based on the weighted data.

**Table 2:** Bullying experiences in relation to school bullying over time between 2009/10 and 2022 (n = 10,556 girls, n = 9,939 boys, n = 124 without gender information) and cyberbullying between 2017/18 and 2022 (n = 5,150 girls, n = 4,597 boys, n = 124 without gender information) Source: HBSC Germany 2009/10, 2013/14, 2017/18 and 2022

Survey year	Uninvolved (in %)	Suffered bullying (in %)	Bully (in %)	Double role bully and bullied (in %)
**School bullying** (X^2^ (9) = 189.7, p < 0.001, V = 0.06, n = 20,619)				
2009/10 (n = 4,910)	81.4_a_	8.6_c_	8.4_d_	1.6_f, g_
2013/14 (n = 5,711)	83.2_a_	7.8_c_	7.5_d_	1.4_f, g_
2017/18 (n = 4,205)	86.7_b_	8.3_c_	3.9_e_	1.1_g_
2022 (n = 5,793)	86.1_b_	8.6_c_	3.4_e_	1.9_f_
**Cyberbullying** (X^2^ (3) = 45.7, p < 0.001, V = 0.07, n = 9,871)				
2017/18 (n = 4,165)	96.0_a_	2.0_c_	1.3_e_	0.6_g_
2022 (n = 5,706)	92.9_b_	3.0_d_	2.7_f_	1.4_h_

Subscripts indicate subgroups that are not significantly different in the post-hoc analyses. Subgroups that do not have the same letter within a bullying role are therefore significantly different from each other. Post-hoc analyses with alpha error correction according to Bonferroni (school bullying: p < 0.008). Values slightly above or below 100 % are due to rounding of decimals. The n in each year refers to the number of cases before weighting. All percentages are based on the weighted data.

**Table 3:** Odds ratios of experiences with school bullying by survey year, gender and age, 2009/10 to 2022 (n = 10,466 girls, n = 9,859 boys) Source: HBSC Germany 2009/10, 2013/14, 2017/18, 2022

	Uninvoled		Suffered bullying		Bully		Double role bully and bullied	
	OR	(95 % CI)	OR	(95 % CI)	OR	(95 % CI)	OR	(95 % CI)
**Survey year** *(Reference: 2022)*								
2009/10	0.4***	(0.4 – 0.6)	1.2	(0.9 – 1.7)	3.3***	(2.3 – 4.8)	2.0	(1.0 – 4.3)
2013/14	0.6***	(0.5 – 0.7)	0.8	(0.6 – 1.2)	3.1***	(2.1 – 4.4)	1.0	(0.5 – 2.2)
2017/18	0.8	(0.6 – 1.1)	1.0	(0.7 – 1.5)	1.9	(1.3 – 2.9)	0.7	(0.3 – 1.7)
**Gender** *(Reference: male)*								
Female	1.3	(1.1 – 1.6)	1.2	(0.9 – 1.5)	0.4***	(0.3 – 0.6)	0.4	(0.2 – 0.9)
**Age** *(Reference: 15 years)*								
11 years	0.8	(0.6 – 1.0)	1.4	(1.0 – 2.0)	0.8	(0.5 – 1.2)	1.6	(0.8 – 3.5)
13 years	0.7	(0.5 – 0.9)	1.4	(1.0 – 1.9)	1.3	(0.9 – 1.9)	2.0	(0.8 – 4.6)

OR = odds ratio, CI = confidence interval

^***^p < 0.001

The significance level was set at p < 0.001 due to the large number of individual comparisons. Results of four logistic regression analyses, one per bullying role.

N refers to the number of cases before weighting. Calculations with weighted data.

**Table 4:** Odds ratios of experiences with cyberbullying by survey year, gender and age in 2017/18 and 2022 (n = 5,110 girls, n = 4,556 boys) Source: HBSC Germany 2017/18, 2022

	Uninvoled		Suffered bullying		Bully		Double role bully and bullied	
	OR	(95 % CI)	OR	(95 % CI)	OR	(95 % CI)	OR	(95 % CI)
**Survey year** *(Reference: 2022)*								
2017/18	2.0**	(1.3 – 3.0)	0.6	(0.3 – 1.2)	0.6	(0.3 – 1.0)	0.4*	(0.2 – 0.9)
**Gender** *(Reference: male)*								
Female	1.7***	(1.3 – 2.3)	1.2	(0.7 – 1.9)	0.3***	(0.2 – 0.5)	0.4**	(0.2 – 0.8)
**Age** *(Reference: 15 years)*								
11 years	1.3	(0.9 – 1.8)	1.2	(0.7 – 2.1)	0.6	(0.4 – 1.0)	0.6	(0.3 – 1.2)
13 years	1.0	(0.7 – 1.5)	1.3	(0.7 – 2.4)	0.9	(0.6 – 1.5)	0.6	(0.3 – 1.2)

OR = Odds Ratio, CI = confidence interval

^*^p < 0.05

^**^p < 0.01

^***^p < 0.001

Results of four logistic regression analyses, one per bullying role. N refers to the number of cases before weighting. Calculations with weighted data.

## References

[1] Olweus D (1994) Bullying at school: basic facts and effects of a school based intervention program. J Child Psychol Psychiatry 35(7):1171–1190 7806605 10.1111/j.1469-7610.1994.tb01229.x

[2] Horton P (2019) School bullying and bare life: Challenging the state of exception. Educ Philos Theory 51(14):1444–1453

[3] Schultze-Krumbholz A Höher J Fiebig J. (2014) Wie definieren Jugendliche in Deutschland Cybermobbing? Eine Fokusgruppenstudie unter Jugendlichen einer deutschen Großstadt. Prax Kinderpsychol Kinderpsychiatr 63(5):361–378 24877777 10.13109/prkk.2014.63.5.361

[4] O’Higgins Norman J (2020) Tackling bullying from the inside out: Shifting paradigms in bullying research and interventions. Int. J. Bullying Prev 2(3):161–169 33005874 10.1007/s42380-020-00076-1 PMC7431550

[5] Fischer SM John N Melzer W. (2020) Traditional bullying and cyberbullying among children and adolescents in Germany – Cross-sectional results of the 2017/18 HBSC study and trends. J Health Monit 5(3):53–68. 10.25646/6902 (As at 24.01.2024) 35146273 PMC8734197

[6] Kochenderfer-Ladd B Ladd GW Thibault SA (2021) School bullying and peer victimization: Its role in students’ academic achievement. In: Smith PK O’Higgins Norman J (Eds) The Wiley Blackwell Handbook of Bullying: A Comprehensive and International Review of Research and Intervention, Vol. 1–2. Wiley Blackwell, Hoboken, NJ, USA, P. 619–638

[7] Heerde JA Hemphill SA (2019) Are Bullying Perpetration and Victimization Associated with Adolescent Deliberate Self-Harm? A Meta-Analysis. Arch Suicide Res 23(3):353–381 29791272 10.1080/13811118.2018.1472690

[8] Moore SE Norman RE Suetani S. (2017) Consequences of bullying victimization in childhood and adolescence: A systematic review and meta-analysis. World J Psychiatry 7(1):60–76 28401049 10.5498/wjp.v7.i1.60 PMC5371173

[9] Midgett A Doumas DM (2019) Witnessing Bullying at School: The Association Between Being a Bystander and Anxiety and Depressive Symptoms. School Ment Health 11(3):454–463 10.1007/s12310-019-09312-6 PMC11101161 38765181

[10] Zych I Ortega-Ruiz R Del Rey R (2015) Systematic review of theoretical studies on bullying and cyberbullying: Facts, knowledge, prevention, and intervention. Aggress Violent Behav 23:1–21

[11] Estévez E Canas E Estevez JF. (2020) Continuity and Overlap of Roles in Victims and Aggressors of Bullying and Cyberbullying in Adolescence: A Systematic Review. Int J Environ Res Public Health 17(20):7452. 10.3390/ijerph17207452 (As at 24.01.2024) 33066202 PMC7602061

[12] Lazuras L Barkoukis V Tsorbatzoudis H (2017) Face-to-face bullying and cyberbullying in adolescents: Trans-contextual effects and role overlap. Technol Soc 48:97–101

[13] Waasdorp TE Bradshaw CP (2015) The overlap between cyberbullying and traditional bullying. J Adolesc Health 56(5):483–488 25631040 10.1016/j.jadohealth.2014.12.002

[14] Baams L Talmage CA Russell ST (2017) Economic costs of bias-based bullying. Sch Psychol Q 32(3):422–433 28661165 10.1037/spq0000211 PMC5578874

[15] Bacher-Hicks A Goodman J Green JG. (2022) The COVID-19 Pandemic Disrupted Both School Bullying and Cyberbullying. Am Econ Rev Insights 4(3):353–370

[16] de Souza VM Levandoski G (2022) Social distancing as a protective barrier against bullying actions among schoolchildren during the COVID-19 pandemic. Work 73(2):383–392 35964227 10.3233/WOR-220160

[17] Borualogo IS Casas F (2023) Sibling Bullying, School Bullying, and Children’s Subjective Well-Being Before and During the COVID-19 Pandemic in Indonesia. Child Indic Res 16(3):1203–1232 36785618 10.1007/s12187-023-10013-5 PMC9907181

[18] Vaillancourt T Brittain H Krygsman A. (2021) School bullying before and during COVID-19: Results from a population-based randomized design. Aggress Behav 47(5):557–569 34235773 10.1002/ab.21986

[19] Da Q Huang J Peng Z. (2023) Did the prevalence of traditional school bullying increase after COVID-19? Evidence from a two-stage cross-sectional study before and during COVID-19 pandemic. Child Abuse Negl 143:106256 37262980 10.1016/j.chiabu.2023.106256 PMC10213298

[20] Barlett CP Simmers MM Roth B. (2021) Comparing cyberbullying prevalence and process before and during the COVID-19 pandemic. J Soc Psychol 161(4):408–418 34128768 10.1080/00224545.2021.1918619

[21] Medienpädagogischer Forschungsverbund Südwest (2023) JIM-Studie 2022. Jugend, Information, Medien. https://www.mpfs.de/studien/jim-studie/2022/ (As at 24.01.2024)

[22] Medienpädagogischer Forschungsverbund Südwest (2023) KIM-Studie 2022. Kindheit, Internet, Medien. https://www.mpfs.de/studien/kim-studie/2022/ (As at 24.01.2024)

[23] Boccio CM Leal WE (2023) Does Socializing in the Virtual World Impact Victimization in the Real World? J Interpers Violence 38(3/4):3756–3776 35866468 10.1177/08862605221109922

[24] Winter K Moor I Markert J. (2024) Concept and methodology of the Health Behaviour in School-aged Children (HBSC) study – Insights into the current 2022 survey and trends in Germany. J Health Monit 9(1):99–117. www.rki.de/jhealthmonit-en (As at 04.03.2024) 10.25646/11878 PMC10977469 38559683

[25] Moor I Winter K Bilz L. (2020) The 2017/18 Health Behaviour in School-aged Children (HBSC) study –Methodology of the World Health Organization’s child and adolescent health study. J Health Monit 5(3):88–102. 10.25646/6904 (As at 24.01.2024) 35146275 PMC8734187

[26] Fischer SM (2021) Self-efficacy, self-regulation, and empathy as parts of teachers’ bullying intervention competence: associations with the intervention behaviour of teachers and the bullying experiences of the students. 10.26127/BTUOpen-5694 (As at 24.01.2024)

[27] Rose CA Espelage DL Monda□Amaya LE (2009) Bullying and victimisation rates among students in general and special education: a comparative analysis. Educ Psychol (Lond) 29(7)761-776

[28] Bucksch J Finne E Gohres H. (2016) Die Methodik des HBSC-Surveys 2013/14. In: Bilz L Sudeck G Bucksch J. (Eds) Schule und Gesundheit Ergebnisse des WHO-Jugendgesundheitssurveys „Health Behaviour in School-aged Children“. Beltz Juventa, Weinheim, P. 35-46

[29] Oertel L Melzer W Schmechtig N (2016) Gewalt und Mobbing im Schulkontext und dessen Folgen für die Gesundheit. In: Bilz L Sudeck G Bucksch J. (Eds) Schule und Gesundheit Ergebnisse des WHO-Jugendgesundheitssurveys „Health Behaviour in School-aged Children“. Beltz Juventa, Weinheim, P. 222-243

[30] Kolip P Hoffarth K Ottova V. (2013) Die Methodik des HBSC-Surveys 2009/10. In: Kolip P Klocke A Melzer W. (Eds) Gesundheit und Gesundheitsverhalten im Geschlechtervergleich. Ergebnisse des WHO-Jugendgesundheitssurvey „Health Behaviour in School-aged Children”. Beltz Juventa, Weinheim Basel, P. 25-35

[31] Oertel L Melzer W Ottova V (2013) Mobbing und Gewalt in der Schule – Unterschiede im Handeln von Jungen und Mädchen. In: Kolip P Klocke A Melzer W. (Eds) Gesundheit und Gesundheitsverhalten im Geschlechtervergleich. Ergebnisse des WHO-Jugendgesundheitssurvey „Health Behaviour in School-aged Children”. Beltz Juventa, Weinheim Basel, P. 145-167

[32] Olweus D (1996) The revised Olweus Bully/Victim questionnaire. University of Bergen/HEMIL

[33] Espino E Jimenez-Diaz O Del Rey R. (2023) Outlining Individual and Contextual Factors Related to LGBTQ+ Bullying: A Systematic Review of Two Decades of Research. Trauma Violence Abuse:15248380231165724. 10.1177/15248380231165724 (As at 24.01.2024) 37078578

[34] Beck D Maranto R Tran B. (2023) Forced choice? Is bullying pushing non-binary students into cyber schools? Educ Rev (Birm):1–11. 10.1080/00131911.2023.2167068 (As at 24.01.2024)

[35] Heino E Ellonen N Kaltiala R (2020) Transgender identity is associated with bullying involvement among Finnish adolescents. Front Psychol 11:612424 33488479 10.3389/fpsyg.2020.612424 PMC7820417

[36] Peguero AA Hong JS (2023) Mobbing in der Schule. Auffälligkeit, Marginalisierung und Viktimisierung von Jugendlichen. Springer, Heidelberg

[37] Schultze-Krumbholz A Göbel K Scheithauer H. (2015) A comparison of classification approaches for cyberbullying and traditional bullying using data from six European countries. J Sch Violence 14(1):47–65

[38] Schultze-Krumbholz A Hess M Pfetsch J. (2018) Who is involved in cyberbullying? Latent class analysis of cyberbullying roles and their associations with aggression, self-esteem, and empathy. Cyberpsychology (Brno) 12(4):2

[39] Bilz L Fischer SM (2020) Interventionsstrategien und Interventionserfolg von Lehrkräften bei Cybermobbing und traditionellem Mobbing aus Schülersicht. Kindheit und Entwicklung 29(2):84–91

[40] Wachs S Bilz L Niproschke S. (2019) Bullying intervention in schools: A multilevel analysis of teachers’ success in handling bullying from the students’ perspective. J Early Adolesc 39(5):642–668

[41] Saarento S Garandeau CF Salmivalli C (2015) Classroom- and school-level contributions to bullying and victimization: A review. J Community Appl Soc Psychol 25(3):204–218

[42] Salmivalli C Lagerspetz K Björkqvist K. (1996) Bullying as a group process: Participant roles and their relations to social status within the group. Aggress Behav 22(1):1–15

